# EGN: a wizard for construction of gene and genome similarity networks

**DOI:** 10.1186/1471-2148-13-146

**Published:** 2013-07-11

**Authors:** Sébastien Halary, James O McInerney, Philippe Lopez, Eric Bapteste

**Affiliations:** 1 Département de sciences biologiques, Institut de recherche en biologie végétale (IRBV), Université de Montréal, Montréal, QC H1X 2B2, Canada; 2 Molecular Evolution and Bioinformatics Unit, Department of Biology, National University of Ireland Maynooth, Co. Kildare, Ireland; 3 Unité Mixte de Recherche Centre National de la Recherche Scientifique7138, Systématique, Adaptation, Evolution, Université Pierre et Marie Curie, 75005, Paris, France

**Keywords:** Similarity network, Evolution, Borrelia, Plasmid, Genomics, Graph, Pathogen, Lateral gene transfer, Introgressive descent

## Abstract

**Background:**

Increasingly, similarity networks are being used for evolutionary analyses of molecular datasets. These networks are very useful, in particular for the analysis of gene sharing, lateral gene transfer and for the detection of distant homologs. Currently, such analyses require some computer programming skills due to the limited availability of user-friendly freely distributed software. Consequently, although appealing, the construction and analyses of these networks remain less familiar to biologists than do phylogenetic approaches.

**Results:**

In order to ease the use of similarity networks in the community of evolutionary biologists, we introduce a software program, EGN, that runs under Linux or MacOSX. EGN automates the reconstruction of gene and genome networks from nucleic and proteic sequences. EGN also implements statistics describing genetic diversity in these samples, for various user-defined thresholds of similarities. In the interest of studying the complexity of evolutionary processes affecting microbial evolution, we applied EGN to a dataset of 571,044 proteic sequences from the three domains of life and from mobile elements. We observed that, in *Borrelia*, plasmids play a different role than in most other eubacteria. Rather than being genetic couriers involved in lateral gene transfer, *Borrelia’s* plasmids and their genes act as private genetic goods, that contribute to the creation of genetic diversity within their parasitic hosts.

**Conclusion:**

EGN can be used for constructing, analyzing, and mining molecular datasets in evolutionary studies. The program can help increase our knowledge of the processes through which genes from distinct sources and/or from multiple genomes co-evolve in lineages of cellular organisms.

## Background

Genomic and metagenomic projects provide an increasing amount of molecular data with a considerable genetic diversity. A portion of these nucleic and proteic data is amenable to standard computationally expensive phylogenetic analyses, through the use of multiple alignments and the construction of individual or concatenated gene phylogenies [1-4]. However, evolutionary analyses of many of these sequences can be carried out using other, less time consuming and more inclusive, approaches [5-8]. Typically, phylogenetic reconstruction is suited for analyzing subsets of homologous genes that can be aligned with confidence. Distant homologs are thus generally absent from these analyses [2]. Moreover, even though classic phylogenetic analyses only require that sequences to be compared are alignable, they often focus on the genealogical relationships between entities from the same level of biological organization (e.g. viruses, plasmids or cellular organisms) resulting from the process of vertical descent. However, gene trees affected by processes of introgressive descent such as lateral gene transfer (LGT) [9-12] pose significant challenges to the reconstruction of a universal tree [13-15] or phylogenetic network of life [16-19]. In particular, it is difficult to study the incongruence between the histories of gene families with uneven distribution among microbial genomes. In addition, it is difficult to represent the transfer of DNA between donors and hosts, while including the vectors responsible for these genetic exchanges on a single representation [8,9]. Viruses (and other mobile genetic elements) are indeed most often not considered to be related to cellular beings, and their evolution as well as that of their genes is generally not described along the organismal species tree [20-22]. Thus some evolutionary information contained in genomic and metagenomic data is not readily exploited in standard phylogenetic analyses.

Consistently, a new suite of methods is becoming increasingly popular, in order to handle more of the complexity of such data. Network-based approaches, that display similarity in a wealth of molecular sequences, have started to offer a valuable complement to improve our evolutionary knowledge on the processes responsible for LGT, as well as on the sources of genetic diversity. They provide useful tools to analyze mosaic sequences [23], genomes harbouring sequences from multiple origins [24-27] and the migration of DNA across metagenomes [28]. Network-based approaches also provide an additional framework in which the genetic diversity of sequences, genomes, or metagenomes can be compared and quantified using graph estimators, even for highly divergent sequences [29]. In general terms, we describe an evolutionary (or similarity) network (to distinguish it from phylogenetic networks) as any graph connecting nodes representing individual sequences, individual genomes or metagenomes, by edges, when these objects present some similarity according to various combinations of operational criteria (e.g. a significant level of similarity between two sequences, as indicated by a BLAST score and/or percentage of similarity; the presence of shared gene families between two genomes; the presence of identical sequences between metagenomes). For the moment, due to the lack of user-friendly freely distributed dedicated software, the construction and analysis of sequence similarity networks requires a certain amount of computer programming skills, and remain less accessible (and therefore less familiar) to biologists than standard phylogenetic approaches.

Here, we introduce a simple but powerful software program, EGN (for Evolutionnary gene and genome network), for the reconstruction of similarity networks from large molecular datasets that may expand the toolkit of evolutionary biologists. EGN is programmed in Perl v5.10.1, it is fast, portable, and runs on Linux and OSX systems. EGN automates the construction of gene and genome networks from nucleic and proteic sequences, coupled to simple statistics describing genetic diversity in these samples, for various user-defined thresholds of similarities. We illustrate some of the options available in EGN, and the novel type of data it exploits. Then, as a proof of concept, we show how EGN can be used to study the complexity of evolutionary processes affecting microbial evolution. We tested whether plasmids were always used as genetic couriers, moving DNA from one lineage to another. Our null hypothesis was therefore that plasmids should always connect to more than one lineage in a gene sharing network in our dataset of 571,044proteic sequences, sampled in genomes from the three domains of life and mobile genetic elements. Our network approaches were able to reject this null hypothesis by identifying a set of plasmids in *Borrelia* that is not being used as such couriers. In this case, plasmids appear to have a different function – that of “evolutionary sandbox”-that contributes to the creation of genetic diversity within their bacterial host lineage.

## Implementation

EGN is implemented in Perl. v5.10.1. The script and a user guide are freely available under the GNU GPL license as Additional file 1 or at http://evol-net.fr. Network construction steps are presented in a simple contextual menu. EGN handles massive datasets of nucleic and/or proteic sequences from FASTA files in DEFLINE format. It automates the identification of homologous sequences using user-defined homology search software (BLAST [30] or BLAT [31]). In short, the identification of similar sequences relies on parameters defining relevant hits (based on e-value, identity thresholds in the aligned regions, minimal hit length), and on parameters tagging the hit quality (such as best-reciprocal hit, minimal length coverage represented by this hit over each of the compared sequences). In EGN, these parameters can be defined by the user. After a step of all against all comparison, clusters of sequences with significant similarities are identified using the exhaustive simple link algorithm [32,33], so that any sequence in a cluster presents at least a significant similarity with another sequence of the cluster, and no similarity with any sequence outside the cluster. Graph-wise, these clusters are called connected components. EGN provides several statistical information for each network as an output file: the average percentage of sequences identity, size (in number of sequences), number of connected components, and a global estimate of the clustering within each component, called graph density, implemented as:

$$G=\frac{2*\mathrm{number}\;\mathrm{of}\;\mathrm{edges}}{\mathrm{number}\;\mathrm{of}\;\mathrm{nodes}*\left(\mathrm{number}\;\mathrm{of}\;\mathrm{nodes}-1\right)}$$

Graph density is comprised between 0 and 1 (i.e *G* reaches 1 when nodes in the component are maximally connected to one another, forming a clique).

The distribution of these connected components in each species/samples is also compiled in a tabulated text outfile. Moreover, EGN produces files that are importable in the popular Cytoscape [34] and Gephi [35] network visualization software programs, in which gene and genomes networks can be further analyzed. EGN also generates FASTA files of sequences in each connected component. These files can be used to generate alignments and standard analyses of selection or phylogenetic analyses. For details, we refer to the User Guide.

## Results and discussion

### EGN analytical workflow

EGN is a script implemented in Perl. v5.10.1 on the Linux and MacOSX platforms for generating evolutionary gene and genome networks from molecular data (proteic and/or nucleic sequences). A simple menu allows users to easily manage the step by step procedure and set up relevant parameters for their analyses. However, BLASTall (v ≥ 2.2.26) [30] or BLAT [35] must be installed on the computer where EGN is executed, and their directory locations properly specified in the OS.

**Figure 1:**
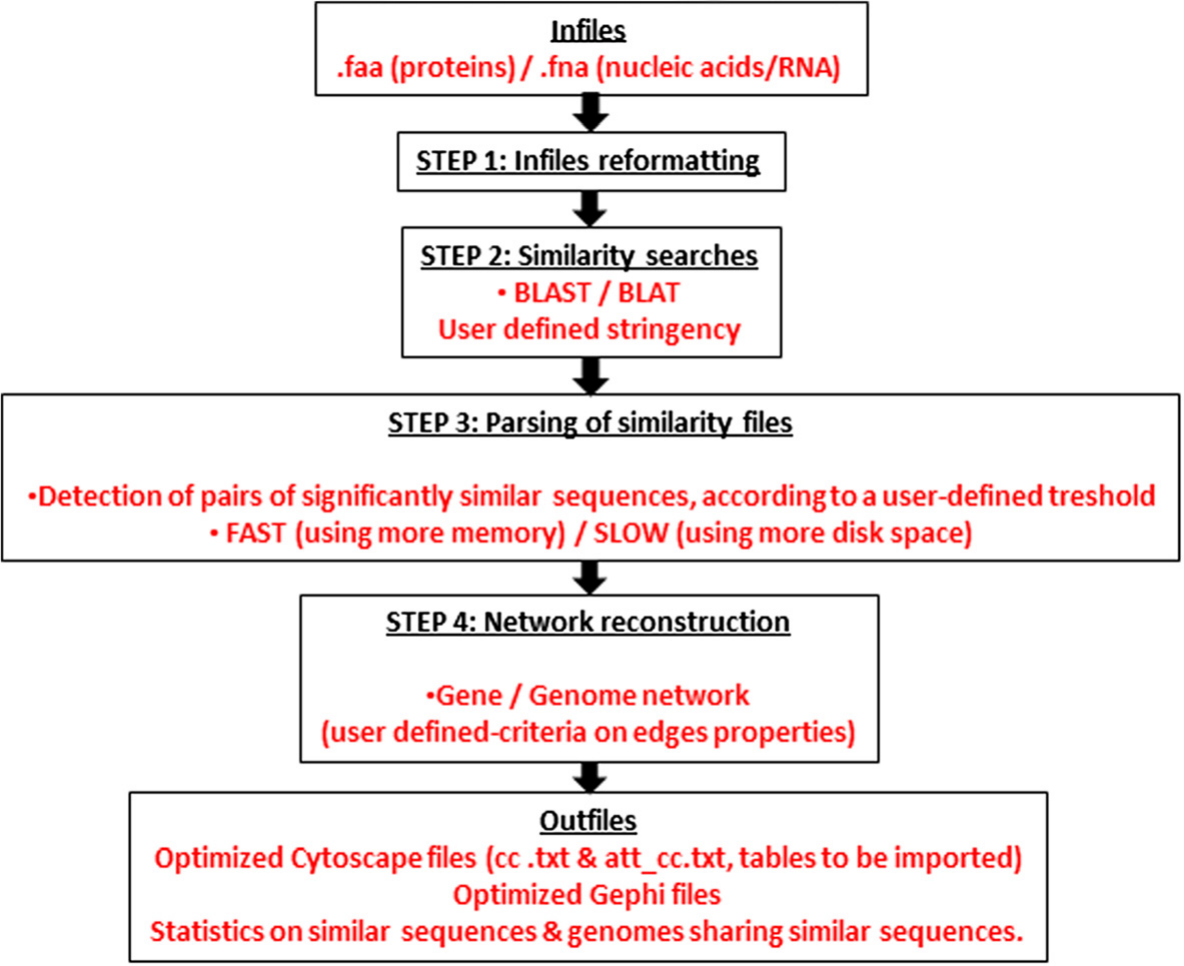
**A schematic of EGN workflow.** This graph represents the different steps achieved in a typical EGN analysis (in chronological order from the top to the bottom of the figure). All options allowing some user-defined choices are indicated in red.

Once EGN is installed, it will take as input one or many files of sequences (in FASTA format) located in a working directory, chosen by the user (e.g. /myEGNanalysis/). The extension of these files must be either .fna, for DNA and RNA sequences, or .faa, for protein sequences. In the case of unique sequence type, user can choose between BLAST or BLAT homology searches to compare these sequences. If the dataset is composed of both nucleic and proteic sequences, BLAST will be chosen and EGN will automatically run BLASTN for nucleic sequences comparison, BLASTP for proteic sequences comparison, while comparisons between nucleic and proteic sequences will be performed by BLASTX and TBLASTN. To this end, EGN must simply be invoked using the command line ‘perl egn.pl’. The software then proposes several analyses, organized in a stepwise fashion (Figure 1).

First, EGN parses the FASTA infiles present in the working directory to i) check that their format is correct (i.e. all sequences have a unique identifier, etc.), ii) to extract useful information about the sequences that will figure in the nodes of the networks under reconstruction (e.g. the sequences/samples/organisms names…), and iii) to assign a local EGN identifier to each sequence in order to speed up the next calculation steps. Once this step of creation of properly formatted input files (option 1, in EGN Main Menu) is successfully achieved, the user can perform two kinds of similarity searches between sequences in these files (option 2 in EGN Main Menu), either by selecting BLASTall [30], or BLAT [31], a faster software program, that speeds up the analysis of very large sequence infiles, but may be less accurate. The user can edit the egn.config file to modify the search parameters of each of these programs or use the wizard implemented in EGN. On multi-core computers, the speed of the BLAST search can be enhanced by changing the number of processors (parameter –a, set to 2 by default). Likewise, we implemented the parallelization of multiple BLAT processes in EGN (default value = 2 in egn.config).

In order to reconstruct a comprehensive similarity network, all sequences can be compared against all. To this end, a third step of EGN (option 3 in EGN Main Menu) parses the results of the similarity search according to a set of properties that any pair of sequences showing similarity must satisfy to be included in a gene or genome network. This parsing step can be optimized since the user can select between two algorithms, depending on the amount of available memory on his/her computer. EGN offers a ‘quicker’ parsing step (option 1, in the Prepare Edge File Menu), requiring a maximum amount of memory, and a ‘slower’ parsing step (option 2, in the Prepare Edge File Menu), needing less memory, but more free disc space.

Under both parsing options, the same large number of desired conditions is available to select relevant sequences and similarity relationships in the Edges File Creation Menu. Option 1 of this menu allows the user to determine a maximal e-value threshold to discard sequences that show too little similarity to be further considered. This decision is facilitated by a simple text interface (default value is also editable in egn.config). Moreover, important additional levels of stringency concerning features of the hit (e.g. the matching sequence segment between two similar sequences) can likewise be specified. Option 2 can be used to impose a minimal percentage of identity over the hit (by default set to 20%). In addition, option 3 can be applied to filter out edges between sequences, when the number of identical bases represents more than a minimal percentage of the shortest sequence length (by default 20%). Option 4 can be used to perform a similar operation since it eliminates edges in which the hit has a minimal absolute length (set by default to 75 for nucleotides and 25 for amino acids, respectively).

Finally, two other properties can also be used to label pairs of similar sequences satisfying the above conditions, and build gene and genome networks. Option 5 evaluates the strength of the similarity between pairs of sequences. In order to determine when two sequences a and b are best-reciprocal hits, EGN computes the e-value of the sequence b in the BLAST (or BLAT) search where sequence a is used as a seed, with the e-value of sequence a in the BLAST (or BLAT) search where sequence b is used as a seed. Sequences a and b may not be each other first best hits in these searches, but when their evalues are no more than a certain used defined percentage away from the top scoring hit in these searches, then sequences a and b are considered as best-reciprocal. By default, this percentage is set to 5. This distinction matters to reconstruct networks based on “best-reciprocal” similarity edges *vs* networks based on any similarity edges, be they “best reciprocal” or not. Option 6 provides a second qualifier for pairs of similar sequences. It allows the user to filter for the extent of the pairwise similarity, i.e. whether a hit spans a great or a small portion of each of the similar sequences. By default, pairs of sequences for which the hit corresponds to ≥ 90% of each sequence length are considered as “homology” edges. Such similarity is not limited to a fragment of any of these sequences, which could happen when significant similarity occurs only for a short region of the sequence, e.g. partial similarity caused by the sharing of a domain.

After these criteria have been set, and the parsing has been carried out, EGN can effectively generate outfiles for two kinds of networks: gene and genome networks (option 4 of EGN Main Menu). This step of network reconstruction also allows the user the option of setting additional selection criteria concerning the edges that will be retained. By default, edges corresponding to hits with a maximal BLAST e-value of 1E-05, 20% identity, and longer than 20% of the smallest similar sequence will feature in gene networks, and be used to reconstruct clusters of similar sequences (see Methods). If two samples/genomes contain sequences belonging to the same cluster, EGN will produce an edge in the genome network between these samples/genomes. In this last construction step, EGN not only generates network outfiles in Cytoscape [34] and/or Gephi format [35], it also optimizes the content of these outfiles. Typically, gene networks may contain hundreds of thousands of subgraphs, corresponding to the cluster of similar sequences, or connected components in graph terms (see next section). Thus, to ease the visualization of these connected components in Cytoscape and Gephi, EGN distributes them in files called (i) cc_#x_to_#y.txt, organizing these subgraphs by decreasing number of edges, and (ii) att_ cc_#x_to_#y.txt, in which all important attributes describing these nodes and edges (e.g. their sample/genome of origin, the weight of edges indicated by the homology searches, whether edges are “full or partial homology” edges, etc.) have been automatically summarized. These attribute files provide useful information for coloring the nodes and edges in the visualization network tools.

Finally, EGN also provides some statistics about the sequences, and the connected components comprising of similar sequences. These text outfiles are created in subdirectories with explicit names, describing the exact parameters retained to perform the network reconstruction (e.g. GENENET_1e-05.50.50.0.0, for the reconstruction of a gene network at 1E-05 e-value threshold, at least 50% hit homology, 50% of homology on the smallest sequence, no best-reciprocal condition, and no minimal coverage condition). In particular, the gpcompo.txt outfile indicates for each cluster of similar sequences how many representative sequences it contains, and from which sample/genome these sequences originate. The gpstat.txt outfile provides further information about genetic divergence of these sequences: how much they cluster with one another in the network, the mean and standard deviation hit % identity between sequences in the cluster, the mean and standard deviation of % identity between the hit and the shortest sequence considered in each pairwise comparison, and the mean and standard deviation of the e-value between sequences of the cluster.

As the user is guided along the various steps of the intuitive EGN menus, this wizard provides a tool with which users will be able to analyze their data under the framework of similarity networks.

### EGN automates the analysis of a significant data type

EGN produces a useful network-based type of data for evolutionary analyses. This data type is different from the usual phylogenetic trees, for at least two reasons. First, while trees are always acyclic graphs, networks are generally cyclic graphs. Second, while phylogenetic trees usually aim at inferring the relationships between homologous sequences and their hypothetical ancestors, sequence similarity networks instead display significant resemblances between any sequences (in gene and protein networks) or any entities (in genome or sample networks), in a topologically less constrained, and in practice much more inclusive, framework. The usual data type used in phylogenetics is a tree (or a grouping on a tree), while it is a connected component in a sequence similarity network. In these latter networks, no explicit orthology relationship needs to be assumed.

It is important to establish the distinction between these two data types, because it would be a logical mistake to evaluate connected components using the standards of phylogenetic trees, e.g. as if they were trees, which they are not. Sequence similarity networks are founded on a different theoretical background than phylogenetic analyses, which implies that the splits and edge lengths have different meanings than those observed in a phylogenetic tree or network. In fact, connected components are better understood by reference to “family resemblances” (a concept brilliantly heralded by Ludwig Wittgenstein, in his posthumously published book Philosophical Investigations of 1953 [36]). Just like family members in humans present various overlapping and criss-crossing resemblances, i.e. in their build, features, colours of eyes, organized in such ways that it is eventually possible and useful to distinguish different families, connected components in sequence similarity networks group sets of sequences, whose members show significant similarity according to a criterion (or a set of criteria), so that these sequences cannot be mistaken for other sequences, presenting a different pattern of “family resemblance”. For instance, sequences coding for translation initiation factors SUI1 and for restriction modification type 1 endonucleases fall into distinct connected components in gene networks [6]. In this regard, it is interesting to note that phylogenetic gene trees are a particular display of one very particular instance of ‘family resemblance’. Such trees group sequences that are sufficiently similar to be aligned together, because they come from a single last common ancestor. However, sequences can also (and not only) display significant similarities that do not meet the particular criteria retained in phylogenetics. For instance, sequences resulting from fusion or recombination events will show *bona fide* similarities introduced by processes of introgressive descent [2]. Sequences evolving by vertical descent from a single ancestor can also become too divergent to be aligned with their homologs, and therefore to be included in a gene tree. Such distant similarities, and resemblances originating from processes of introgressive descent, however can be analyzed through the definition of connected components, as automated by EGN. For example, when the parameters selected for the reconstruction of the gene networks are very stringent, imposing that the hit between sequences covers a high percentage of their length, and that the similar sequences show both a high e-value and % identity, allows to include divergent homologs in connected components. Unlike conserved homologs that will be all connected together (forming a pattern of maximal density known as a clique in the connected component), divergent homologs will only connect to some of the sequences within the component; i.e. divergent bacterial genes will only bond to some of their bacterial homologs, while less divergent bacterial genes will all be joined to one another.

Consistently, the data type that is obtained by structuring molecular data in connected components of sequences in sequence similarity networks (or in connected components of genomes sharing similar sequences in genome networks), contributes in a different way than phylogenetics to extend the scope of evolutionary analyses. Evolutionary biologists can take advantage of this additional data type to explore and explain the various causes of their ‘family resemblances’. Processes of introgressive descent (e.g. recombination, lateral gene transfer, gene or domain sharing, etc.) and vertical descent can be investigated simultaneously through these graphs. Phylogenetic relationships however will generally still require the reconstruction of a tree. Furthermore, this novel data type also provides an original comparative framework, which must not be confused with the phylogenetic framework. More precisely, EGN networks make it possible to compare sequence similarities for sequences of interest in connected components, i.e. by quantifying the distances and topological properties of sequences from two genomes in the network. This comparison cannot be equated with the phylogenetic resolution required to identify where a particular sequence (or organism) should be placed in a gene (or organismal) tree, but it can be useful in other situations. Among the most recent examples, a comparative analysis of the behavior of sequences in gene networks was carried out by Bhattacharya et al. [29] to investigate the modular genomic structure of a novel marine cyanophage in a protist single cell metagenome assembly. Sequences from these novel mosaic viruses presented a pattern of connection that was typical of that presented by sequences from mosaic cyanophages in the gene network. The use of a network proved particularly well-suited, offering much more detail concerning the complex evolution of such mosaic objects than allowed by the proposition of a single branching point in a viral tree for the novel virus.

One main interest of network studies is therefore that they can employ an additional, very inclusive, relevant - although non-phylogenetic - data structure for evolutionary analyses.

### Application to real data

We used EGN to illustrate how its various options areuseful for devising and testing evolutionary hypotheses, while taking into account a large amount of data structured according to this data type. We tested whether plasmids were always used as genetic couriers, moving DNA from one lineage to another in a dataset of 571,044 protein sequences (see Implementation). We first used EGN (e-value ≤ 1E-20, hit identity ≥ 30% ) to reconstruct a genome network of 131 cellular organisms, 2,211 plasmids and 3,477 viral genomes, harboring either cellular chromosomes or genomes of mobile genetic elements at its nodes, connected when they shared sequences from the same similarity cluster, as in Halary *et al.*[25].

**Figure 2:**
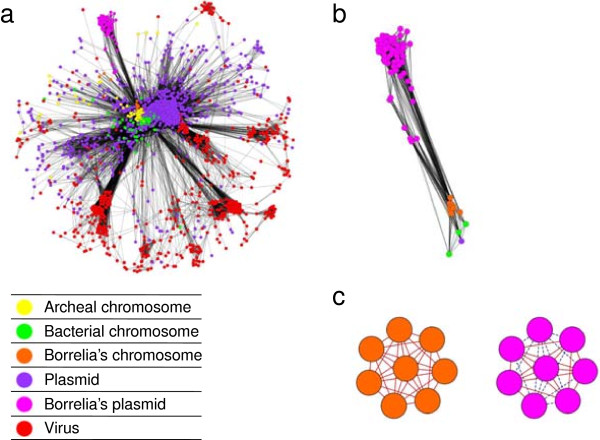
**Networks reconstructed using EGN. a**. Principal connected component of the genome network (e-value ≤ 1E-05, identities ≥ 30%, with “best reciprocity” option). Node colors are reported below the component. *Borrelia*’s plasmids (pink) are tightly packed together and relatively isolated from the rest of the network. **b**. Detail of the genome network showing only nodes linked with *Borrelia*’s plasmids. *Borrelia*’s plasmids are directly only connected to *Borrelia*’s chromosomes. **c**. Schematic connected components, same color code as above. “Full homology” edges are indicated by solid lines, other similarity edges are indicated by dashes. Components with a majority of nodes corresponding to genes on chromosomes were significantly richer in “full homology” edges than connected components with a majority of nodes corresponding to genes on plasmids.

In the genome network, some plasmids displayed markedly distinct behaviors and patterns of connections, identifying two extreme sorts of plasmids. On the one hand, many plasmids had a broad range of connections with a diversity of distantly related genetic partners. These plasmids act as genetic couriers [37], contributing to exchanges of DNA material. On the other hand, some other plasmids were very isolated in the network, showing a very limited and sometimes even no genetic partnerships outside a limited gene sharing with the plasmids or the chromosomes of their host lineage. Plasmids of this second type typically use a closed DNA pool, and seem to rarely transit between different hosts cells and lineages, and even to rarely exchange genetic material with the chromosome of their hosts. Rather than being mobile vessels of genetic exchange, our network suggests that these non-promiscuous plasmids may fulfill a functional role of evolutionary significance distinct from that of the plasmids that are key players for lateral gene transfer. The best examples are offered by the plasmids of the bacterial genus *Borrelia*, which display a very low conductance (C = 0.015). Indeed, the corresponding/respective nodes are extremely isolated in the genome network, and are linked to it just by edges with nodes of the *Borrelia* chromosomes (Figure 1). *Borrelia*’s plasmids do not share a single gene family with any other plasmids outside these hosts (Figure 2a), and only harbor six genes (*oligopeptide ABC transporter, vlp protein alpha and gamma subfamily, arginine-ornithine antiporter, putative lipoprotein* and *type I restriction enzyme R protein*), that are also found on *Borrelia*’s main chromosome (Figure 2b), consistently with the literature [38]. In agreement with Tamminen *et al.*[39], our genome network identifies that the flow of DNA material in and out of *Borrelia* plasmids is lower than for many other plasmids.

This remarkable genetic isolation may be explained by biological considerations, prompted by the detection of this network structure. *Borrelia* is an obligate pathogen [40]. This lifestyle entails that these bacteria have fewer opportunities to meet a diversity of genetic partners (be they mobile elements or other bacteria) than the majority of the bacteria growing in biofilms [41]. *Borrelia* genetic diversity must come from within the lineage, rather than from adaptative gene transfer from other microbes, even though *Borrelia*’s plasmids are able to transfer [42,43]. Moreover, *Borrelia*’s lifestyle imposes a strong selective pressure on these parasitic cells that must constantly evolve to escape their host immune system. Plasmids within *Borrelia* play a role in this evasion process [44-47], and we hypothesize that it is because they provide a genetic compartmentalization inside the cells that allows *Borrelia* to partition DNA on two distinct kinds of molecules with distinct evolutionary regimes [48,49]. Most of the genes are located on a slow evolving linear chromosome, heavily constrained in its structure, while other genes are stored on the more flexible, fast evolving, and heavily recombining plasmids[43,49,50]. We propose that this partition helps *Borrelia* cells to survive in a hostile environment. The chromosomes are highly streamlined and optimized to support *Borrelia*’s parasitic life, while the plasmids would be the locus of substantial rearrangements, recombination and gene conversion, producing necessary variations on genes coding for outer surface proteins (*osp*), genes that repress the cytolitic activity of host’s serum (*Erp* and *CRASP*), and genes coding for antigenic variation (*vlp*, *vsp*, *vlsE*) to escape *Borrelia*’s host immune system [40,44-47].

To further this hypothesis - that some plasmids act as compartments of DNA material and intracellular organs of genetic innovation rather than vectors of mobile DNA - we used the hit coverage option of EGN’s gene network reconstruction, that we set to > 90%. This option allowed us to distinguish two types of edges in sequences network. We used EGN to detect and quantify “full homology” edge links between *Borrelia*’s sequences. Sequences connected by “full homology” edges have probably progressively diverged by the combined effects of small mutations, natural selection and drift, because their sequences greatly overlap, and can be aligned all along their length. By contrast, when sequences are not only evolving in a tree-like fashion, i.e. when segments of divergent sequences fuse to form a single a gene, or when segments within genes recombine through illegitimate recombination, sequences are connected by edges that are not necessarily “full homology” edges [23]. These sequences do not come entirely from a single ancestral gene copy, but various segments of these sequences have a diversity of sources. Such sequences, produced by more complex processes than vertical descent alone, do not neatly align all along their sequences, but are at best only connected through local regions of similarity. Such similar segments, as opposed to similarity overall their DNA, are also detected in EGN analyses: they constitute a second type of edge in gene networks (Figure 2c). Interestingly, we observed that connected components of *Borrelia’s* chromosomes were largely connected by “full homology” edges (thus likely evolving by vertical descent), but that connected components on *Borrelia*’s plasmids were largely connected by “partly similar edges”, and therefore seemed to be subjected to more complex evolutionary processes. These processes result in a large amount of genetic diversity in the plasmids.

This result, based on a gene network, (Figure 2b) strengthens our hypothesis that a structural partition of DNA within *Borrelia*’s cells (observed in the genome network) is coupled with a “partition” of the processes affecting this DNA, in that case contributing to the recruitment of *Borrelia*’s plasmids as “organs of genetic innovation”. In other words, *Borrelia*’s plasmids and their genes can be seen as private goods of the *Borrelia* lineage [7]: they benefit to this lineage but are not shared with others. Of course, quite a few other prokaryotic genera contain plasmids with low conductances, such *Sodalis*(C = 0.16), *Coxiella* (C = 0.17)*,* and *Buchnera* (C = 0.23) (Additional file 2: Table S1 & Additional file 3: Figure S2, see Implementation). We do not wish to elaborate here on whether the lifestyle of these bacteria may explain this relative genetic isolation. *Sodalis* are intra and intercellular symbionts, *Buchnera* are obligate intracellular symbionts and *Coxiella* are obligate intracellular pathogens. However, we want to underscore the fact that sequence similarity network can be a great tool to foster this type of hypothesis.

## Conclusions

The use of similarity networks appears as a compelling complement to standard phylogenetic analyses in order to perform comparative analyses of an increasing amount of molecular sequences from genomic and metagenomic projects. Several publications have already benefited from this analytical framework [2,6,25,29,51,52]. However, such network analyses still require more programming skills than is usually necessary to carry out phylogenetic analyses, for which users can rely on a diversity of user-friendly software. By contrast, few (if any) user friendly software programs, running on desktop computers, explicitly designed to reconstruct distinct kinds of similarity networks from nucleic and/or proteic data have yet been made available to the biology community. We introduce EGN in the hope that it might constitute a timely opportunity to provide network construction tools to a broader audience. We are confident that software like EGN will enhance the exploitation of the evolutionary signal of genomic and metagenomic projects.

### Genomic datasets and analysis parameters

We sampled 571,044 protein sequences from the chromosomes of 70 eubacterial complete genomes, 54 archaebacterial complete genomes, and 7 eukaryotic genomes, covering the diversity of cellular life, as well as from the genomes of two types of mobile genetic elements: 228,040 protein sequences from all the available plasmids and phages at the time of this analysis from the NCBI (see Additional files). We first used EGN (e-value cutoff 1E-05, identity thresholds 30%) to construct a genome network, harboring either cellular chromosomes or genomes of mobile genetic elements at its nodes, connected when they shared sequences belonging to the same cluster of similar sequences, as in Halary *et al.*[25]. To test whether plasmids hosted in a bacterial lineage were connected to genomes in multiple other lineages, we estimated the conductance of their nodes (C) in the genome network. For instance, for plasmids of *Borrelia*, we estimated C as the number of edges connecting *Borrelia*’s nodes to non-*Borrelia*’s nodes / number of edges connecting *Borrelia*’s nodes to any node [53]. We assessed whether the observed value for C was significantly different and lower than the conductance obtained by chance for the same number of nodes in the genome network by shuffling node labels on the same network topology for 1,000 replicates, which estimates the various conductances expected by chance alone in a network of same size and with the same topology.

In order to test whether genes within *Borrelia* chromosomes and plasmids had been affected by different evolutionary processes, we then reconstructed the similarity network of *Borrelia* genes using the same parameters, but setting up the hit coverage condition at > 90% (e.g. edges were tagged as positive when the hit was longer than 90% of each gene’s length, else negative). Number of ‘positive’ and ‘negative’ edges linking plasmidic gene to plasmidic gene, plasmidic gene to chromosomal gene, and chromosomal gene to chromosomal gene were quantified. Over- or under- representation of such edges was also estimated by shuffling node labels on the same network topology for 1,000 replicates.

## Availability and requirements

•**Project name:**EGN

•**Project home page:**http://www.evol-net.fr/index.php/fr/downloads

•**Operating system(s):** Linux Platform and OSX

•**Programming language:** Perl. v5.10.1

•**Other requirements:** BLAST + versions ≥ v2.2.25 or BLAST all versions ≥ v2.2.26 or BLAT

•**License:** GNU GPL

•**Any restrictions to use by non-academics:** none

### Availability of supporting data

The dataset supporting the results of this article is available in the repository of http://www.evol-net.fr/index.php/fr/downloads. The EGN script and a user guide are also available at this address and as Additional file 1.

## Abbreviations

EGN: Evolutionary gene and genome network; C: Conductance.

## Competing interests

The authors declare that they have no competing interest.

## Authors’ contributions

EB, PL and SH designed the software. SH implemented the software. EB, PL and JMI designed the experiment. EB, PL, JOM and SH analyzed the results. EB, JOM, and SH wrote the paper. All authors read and approved the final manuscript.

## Supplementary Material

Additional file 1 A zip archive containing the EGN script and a user guide. Click here for file

Additional file 2: Table S1 An xls table including the summary of the conductance analyses for all plasmids and plasmids of all the prokaryotic genera present in our analyses. Click here for file

Additional file 3: Figure S1 A tif file with the graphical representation of the conductance analyses for all plasmids and plasmids of all the genera present in our analyses. Y-axis corresponds to the conductance value (C) of these plasmids. Each dot corresponds to a class of plasmids. Dots colored in red, black, green and purple correspond to plasmids hosted in *Borrelia*, *Sodalis*, *Coxiella* and *Buchnera*, respectively. Click here for file
