# Supplementary material for: EGN: a wizard for construction of gene and genome similarity networks
Source: BMC Evol Biol. 2013 Jul 11;13:146. doi: 10.1186/1471-2148-13-146 (PMC3727994; doi:10.1186/1471-2148-13-146)
Supplement: Additional file 1 — A zip archive containing the EGN script and a user guide. [file 1471-2148-13-146-S1.zip › EGN/EGN User Guide.pdf]

EGN User Guide

Evolutionary Gene and Genome Networks generator

October 2012 / version 1.0

Contact: sebastien.halary@umontreal.ca

Citation:

EGN: a wizard for construction of gene and genome similarity networks.

SebastienHalary, James McInerney, Philippe Lopez , Eric Bapteste

Table of contents

1. Pre-requisites .... - 4 - ~~- 2 -~~

2. Installation .... - 4 - ~~- 3 -~~

3. Quick start .... - 4 - ~~- 3 -~~

4. EGN Menu .... - 5 - ~~- 3 -~~

4.1 Create the input files .... - 5 - ~~- 3 -~~

4.1.1 Fasta format .... - 5 - ~~- 4 -~~

4.2 Similarity search in sequences .... - 6 - ~~- 4 -~~

4.2.1 *egn.config* file .... - 6 - ~~- 4 -~~

4.2.2 BLAT or Blast .... - 6 - ~~- 5 -~~

4.3 Prepare Edges File .... - 6 - ~~- 5 -~~

4.3.1 Quicker or Slower Algorithm .... - 6 - ~~- 5 -~~

4.3.2 Edges parameters .... - 7 - ~~- 5 -~~

4.4 Networks .... - 7 - ~~- 6 -~~

4.4.1 Gene network .... - 7 - ~~- 6 -~~

4.4.2 Genome network .... - 9 - ~~- 7 -~~

References .... - 10 - ~~- 9 -~~

EGN is a perl script which eases the generation of evolutionary gene and genome similarity networks from molecular data (proteic and/or nucleic sequences) stored in fasta files. The contextual menu allows to easily manage the step by step procedure and to set up parameters.

1. Pre-requisites

EGN script was implemented in Perl. v5.10.1 on the Linux platform.

BLASTall (≤v. 2.2.26) (ftp://ftp.ncbi.nih.gov/blast/executables/release/2.2.26/), BLAST+ (≤v. 2.2.25) (ftp://ftp.ncbi.nih.gov/blast/executables/blast+/) or BLAT (http://hgdownload.cse.ucsc.edu/admin/exe/) must be installed on the computer and properly mentioned in the OS path.

Code de champ modifié

To set the PATH variable, you can either type in your terminal:

export PATH=/full path of your BLAST and/or BLAT binaries directory/bin:$PATH

This path will be effective until you close the terminal window, or better that you modify it permanently, by editing the .profile file (or the .bashrc file, depending of the terminal's type that you use) localized in your home (Linux) or Users (Mac OS) directory:

echo 'export PATH=/full path of your BLAST and/or BLAT binaries directory/bin:$PATH' >> .profile

This command line will also create the .profile file in the case it doesn't exist before. The new path will be permanently effective after the opening of a new terminal window.

2. Installation

Download an archive file egn.tar or egn.plus.tar, depending on the BLAST version you want to use. EGN (egn.tar) is compatible with BLASTall and was tested for the BLASTall versions ≤ v2.2.26, EGN.plus (egn.plus.tar) with BLAST+ versions ≤ v2.2.25.

Decompress it using the command line:

tar –xvf egn.tar

3. Quick start

Create a working folder (e.g. using command 'mkdir myEGNanalysis') and place the sequence fasta file(s) in this folder.

If necessary, rename fasta files with .fna or .faa extensions depending of their sequence type (e.g. *.fna for DNA/RNA; *.faa for proteins).

Put this folder as current folder (e.g. using command 'cd myEGNanalysis') and start EGN by typing:

perl egn_directory_path/egn.1.0.pl

4. EGN Menu

The pipeline consists in 4 steps, each of them creating output files which will be used by the next step. Then, EGN can be stopped between 2 steps, and the process restarted later.

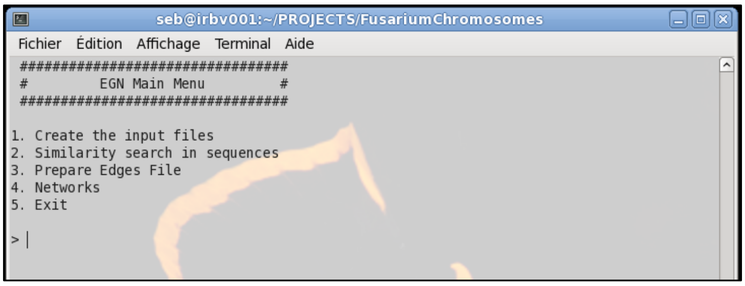

Screen capture of the main menu

4.1 Create the input files

EGN parses the fasta infiles present in the working directory to extract useful information about the sequences for labeling nodes in the network (i.e. the sequences/samples/organisms names) and to assign a local identifier to each sequence in order to speed up the next calculations steps. In this aim, a specific fasta header format is needed.

4.1.1 Fasta format

Sequences files must be formatted following fasta DEFLINE conventions. Sequences downloaded from genbank or embl (for instance) are thus directly usable.

Example: **>tag|seqID**|optional information [sample/organism] #attribute

In bold: essential elements

- tag must be a word ([a-z] or _). Typically gi, emb,ref, or lcl.
- seqID may contain [a-z], [0-9], "." and "_".
- optional information may contain any character except "#", "[" and "]".
- sample/organism's name may contain any character except "#", "[" and "]" but must be surrounded by "[" and "]". An organism or sample name can provide useful information for gene networks composition and visualization, and are required for genome networks.
- attribute may contain [a-z], [0-9], "." and "_" and must be placed after a #. Attributes can be used to add complementary informations about the sequence, as environment's type or host's name. This information will be used as Boolean parameters.

Sample/organism's names and attributes will be used to label nodes in the reconstructed network. Even if the optional information are not stored, the original header of the sequence will be retrieved in the final fasta output files of the homolog sequences groups.

If a sequence’s header format is incorrect, the parsing is stopped to display the line and the name of the problematic sequence and file.

4.2 Similarity search in sequences

To compare large sequences batch, BLAST [1] and BLAT [2] have been chosen. At this step, EGN acts as a wrapper to run the appropriate sequence of command lines, using software parameters values editable in the *egn.config* file.

Code de champ modifié

Code de champ modifié

4.2.1 *egn.config* file

All parameters values used by EGN can be edited the egn.config. Descriptions of each parameter are copied from the manual of BLAST and BLAT.

You must notice that:

- Only BLAST parameters which are common to BLASTN, BLASTP, TBLASTN and BLASTX are settable.
- You can change the BLAST number of database sequences to show one-line (–v or -num_descriptions) value to modify the maximum number of possible edges by node.
- Place a # at the beginning of a line to comment it if needed. It could be useful if you don’t want to use the -ooc BLAT parameter. Comment the corresponding line will make the alignment slower but more sensitive, as explained at http://genome.ucsc.edu/FAQ/FAQblat.html.
- EGN is able to launch several BLAT processes at the same time. You can choose the number of processes by editing the “#Number of processors” line. This could highly impact the RAM usage.

4.2.2 BLAT or BLAST

BLAT was chosen for its ability to quickly align a large number of sequences. But its application is limited to a single type of sequences. Then, if you want to create a network from nucleic, or proteic sequences only, BLAT seems to be a good option.

BLAST has to be used when your dataset is composed by nucleic and proteic sequences. In the aim to compare all sequences together, EGN will run BLASTN (n/n), BLASTP (p/p), BLASTX (n/p) and TBLASTN (p/n) sequentially.

4.3 Prepare Edges File

This step consists in parsing alignment output files to hold only hits presenting identity values according to user-determined thresholds, which will be considered as edges in the network. When there are two hits, for a couple of sequences (a→b and b→a), EGN will keep the best-scored hit.

4.3.1 Quicker or Slower Algorithm?

The quicker option will store in RAM each hit a→b until it finds the mirrored one b→a, before detecting the best one and calculate identity values to determine whether the hit will define an edge. Depending on your dataset, it could require a lot of RAM. Therefore the slower alternative that divides the process in sub-tasks, and writes temporary files in the hard drive was also implemented.

4.3.2 Edges parameters

- E-value threshold: maximal hit's e-value to consider the creation of an edge. (The default value of the menu was extracted from *egn.config*).

- Hit identity threshold: minimal hit's identity value to consider for the creation of an edge. This value is directly extracted from the BLAST/BLAT output file.

- Hit length % of the shortest homolog: Minimal value of the relative hit identity calculated using the formula:

$$\frac{\text{number of identical residues}}{\text{shortest sequence length}} * 100$$

as described [3].

Code de champ modifié

- Minimal hit length: minimal length of the hit to consider for the creation of an edge. This length is expressed in nucleic acids and will be divided by three by the software in the case of protein sequences comparison.

- Best-reciprocity: The edge a/b will be tagged as best-reciprocal if the e-value of the a→b hit fell into the range between the best hit e-value of the query a (BHEa) and BHEa+(X% of BHEa) AND if the evalue of b→a hit fell into [BHEb; BHEb+(X% of BHEb)]. This option (e.g. X = 5) can be useful to construct networks with closely related sequences.

- Hit coverage: This option allows taking into account only hits representing X% of the length of both sequences. For instance, a high hit coverage threshold can discard edges resulting from a recombination event in one of both sequences, or similarity only due to the sharing of a domain.

4.4 Networks

From the edges list, EGN is able to create gene or genome networks. The output files are stored in directories called GENET or GENOMNET depending on the networks type. This directory name also includes 5 values of edges parameters (E-value, Hit identity, Hit identity wrt the smallest sequence, Best-reciprocity option used (0/1), Hit coverage option used (0/1)). Note that EGN allows the user to create a new edges filter by changing the identity threshold values in that menu. The default values displayed by the menu then correspond to those used in the previous step. This was implemented to allow the user to refine its network's properties without re-compute the time and RAM expensive edge preparation step. Two other options are also available at this step: to filter edges to only keep those corresponding to Best-Reciprocal hits, and to tag edges fulfilling the hit coverage condition.

4.4.1 Gene network

In gene networks, nodes represent genes and edges represent a relevant similarity between two nodes. In the network visualization software, the user will choose to represent the length of an edge as the inverse proportion of the hit identity (%), the shortest sequence identity (%) or the e-value. This

network is constructed from the edges list using a simple linkage algorithm, which has the advantage to be exhaustive. EGN can provide 5 types of output:

- Groups statistics: this tabulated file *gpstat.txt* summarizes statistical information about groups, corresponding to connected components, sorted from largest to the smallest component, and trransitivity, given by the formula: $t = \frac{2* number\ of\ edges}{number\ of\ nodes*(number\ of\ nodes\ -1)}$.

Group	Sequence number	Transitivity	Mean hit identity (%)	SD hit identity (%)	Mean shortest sequence identity (%)	SD shortest sequence identity (%)	Mean E-value	SD E-value
**1**	69	0.00	86.48	6.22	37.27	18.19	2e-13	2e-11
**2**	62	0.01	85.25	8.83	36.70	18.84	1e-20	2e-19
**3**	40	0.08	84.29	7.60	44.32	19.42	3e-17	5e-16

Example of gpstat.txt

- Groups composition information: *gpcompo.txt* is a tabulated file summarizing the number of nodes by group belonging to each sample/organism, and tagged by each attribute.

Group	Sequence number	Borrelia afzelii PKo	Borrelia burgdorferi ZS7	Borrelia duttonii Ly	Borrelia garinii PBi	Borrelia hermsii DAH	Germany	Tanzania	USA
**1**	69	12	14	16	17	10	43	16	10
**2**	62	4	12	16	18	12	34	16	12
**3**	40	8	7	9	11	5	26	9	5

Example of gpcompo.txt, where attributes correspond to country of the strain isolation.

- Cytoscape inputs: EGN creates a CYTOSCAPE directory containing two file types.

- cc_x.to.y.txt are files containing the connected components numbered from x to y, and importable in Cytoscape. The cc*.txt file is organized as a table, that contains information about the nodes connected in the network, and various weights for the corresponding edges. To be visualized, the cc*.txt file must be imported in Cytoscape using the File menu, option Import, sub-option Network from Table (Text/MS Excel). The first line describes what is in each column, generally: ID1, ID2, HIT%ID, SS%ID, E-value, MIN_COV_OPT (1 for edges between sequences showing a higher hit coverage than the user defined treshold, 0 otherwise). This first line must be discarded by ticking the box 'Show Text File Import Option', and then by ticking the command 'Transfer first line as attribute names' in the Attribute Names section in Cytoscape. Then, each line describes an edge between two nodes (seq1 and seq2) in the following format, using tabulation as space delimiters:

<EGN number of seq1> <EGN number of seq2> <HIT%ID> <SS%ID> <E-value> <MIN_COV_OPT>

The first and second columns must thus be selected as in the section Interaction Definition. Source interaction must read Column 1, and Target interaction must read Column 2 in Cytoscape, and one weight can be selected if desired (note that this latter selection is not mandatory). Users must then select Import, and go into the Layout menu to display the graph (option Cytoscape layouts). We generally recommend the use of the Force-Directed layout (weighted or unweighted). Nodes and edges can then be colored in the control panel, in the VizMapper menu, according the cytoscape manual (to be consulted for more information).

A connected component file does not contain more than 150 000 edges to allow to Cytoscape to display them without too important computer slowdown. User will be able to

choose between the hit identity, identity on the shortest sequence and E-value to set-up the edges length. Edges can also be characterized by their hit coverage.

- att_cc_x.to.y.txt are files containing samples and attributes information to color networks nodes. These nodes and edges attributes generated by EGN in the att_cc*.txt file must be uploaded in Cytoscape using the File menu, and the Import option, and Attribute from Table (Text/ MS Excel) sub-option. The first line of this file describes what is in the column (generally ID and SAMPLE), and must be discarded using the Show Text File Import Options, and the Transfer first line as attribute names sub-option. Once they are selected, attributes can be simply associated to the network using the Data Panel Menu, option Select Attributes.

- Gephi inputs: EGN can also generate *.gexf* inputs for Gephi stored in the GEPHI directory, which contain the same information than cc_ and att_ cytoscape inputs files. As we noticed serious slowdown when Gephi displayed more than one connected component, EGN provides one file by cc. Gephi option is rather to be used for genome networks or very large connected components.

- Fasta Files: In the aim to allow further analyses, EGN can provide fasta files of sequences for each connected component. The header of the sequence will be the same than those of the inputs provided by the user.

4.4.2 Genome network

EGN is able to provide genome networks, where nodes are Sample/Organism and edges inversely proportional to the number of connected components to which the 2 two nodes collectively belong. Two outputs can be obtained:

- Cytoscape inputs: see corresponding section in Gene Network.
- Gephi inputs: see corresponding section in Gene Network.

References

1. Altschul SF, Madden TL, Schaffer AA, Zhang J, Zhang Z, Miller W, Lipman DJ: **Gapped BLAST and PSI-BLAST: a new generation of protein database search programs**. *Nucleic acids research* 1997, **25**(17):3389-3402.
2. Kent WJ: **BLAT--the BLAST-like alignment tool**. *Genome research* 2002, **12**(4):656-664.
3. Raghava GP, Barton GJ: **Quantification of the variation in percentage identity for protein sequence alignments**. *BMC bioinformatics* 2006, **7**:415.
