# Supplementary figures and images for: EGN: a wizard for construction of gene and genome similarity networks
Source: BMC Evol Biol. 2013 Jul 11;13:146. doi: 10.1186/1471-2148-13-146 (PMC3727994; doi:10.1186/1471-2148-13-146)

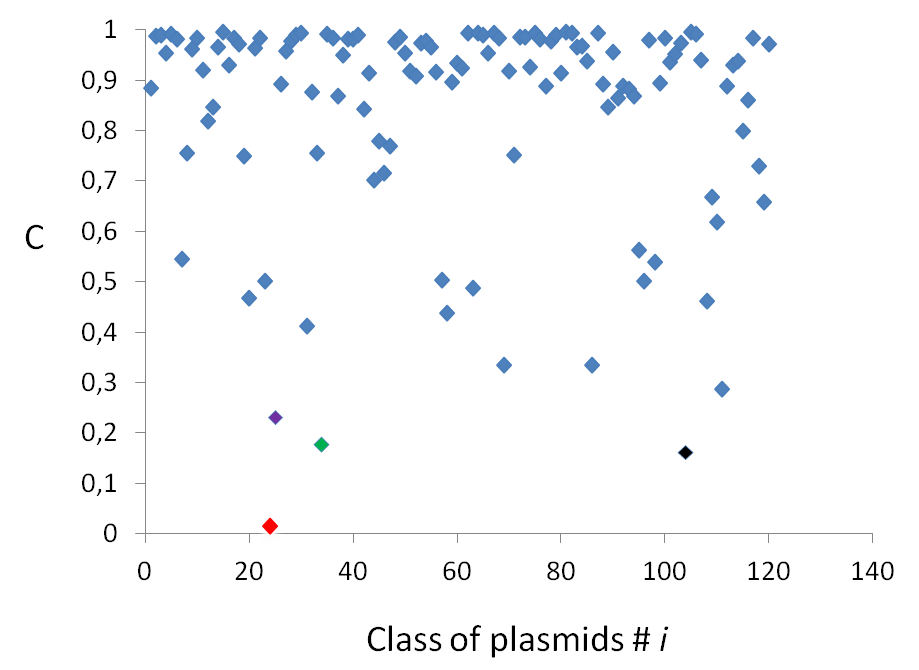

Supplement: Additional file 3: Figure S1 — A tif file with the graphical representation of the conductance analyses for all plasmids and plasmids of all the genera present in our analyses. Y-axis corresponds to the conductance value (C) of these plasmids. Each dot corresponds to a class of plasmids. Dots colored in red, black, green and purple correspond to plasmids hosted in Borrelia, Sodalis, Coxiella and Buchnera, respectively. [file 1471-2148-13-146-S3.tiff]
